# Reliability and correlates of intraindividual variability in the oculomotor system

**DOI:** 10.16910/jemr.12.6.11

**Published:** 2019-10-02

**Authors:** Marlou Nadine Perquin, Aline Bompas

**Affiliations:** CUBRIC, Cardiff University, United Kingdom

**Keywords:** Eye movement, eye tracking, microsaccades, gaze, attention, reliability, intraindividual variability, individual differences, ADHD, mind wandering

## Abstract

Even if all external circumstances are kept equal, the oculomotor system shows intraindividual variability over time, affecting measures such as microsaccade rate, blink rate, pupil size, and gaze position. Recently, some of these measures have been associated with ADHD on a between-subject level. However, it remains unclear to what extent these measures constitute stable individual traits. In the current study, we investigate the intraindividual reliability of these oculomotor features. Combining results over three experiments (> 100 healthy participants), we find that most measures show good intra-individual reliability over different time points (repeatability) as well as over different conditions (generalisation). However, we find evidence against any correlation with self-assessed ADHD tendencies, mind wandering, and impulsivity. As such, the oculomotor system shows reliable intra-individual reliability, but its benefit for investigating self-assessed individual differences in healthy subjects remains unclear. With our results, we highlight the importance of reliability and statistical power when studying between-subject differences.

## Introduction

Imagine that you are working in your office, and one of your colleagues suddenly walks in: Your eyes will immediately change position from your work to your colleague, and your pupil size will be modulated by the differences in light hitting your eye. These changes in eye position and pupil size may be described as ‘exogenously-driven’ – variability within an individual over time that is brought about by changes in the external environment. However, even when all external circumstances remain the same and one is solely fixating on a static dot, the oculomotor system still shows variability, such as fluctuations in eye position (i.e., ‘fixational eye movements’, see (1) for a review) or pupil size, and blinks. All of these changes may be described as ‘purely endogenous’ intra-individual variability – brought about by internal fluctuations. Oculomotor variability measured during a psychophysical task will reflect both exogenous and endogenous fluctuations, and quantifying their respective contributions would be difficult. Alternatively, endogenous or ‘basic’ activity can be measured in ‘resting-state based paradigms’, during which the environment is kept stable for a prolonged period of time. Such resting-states have gained popularity in neuroimaging studies, but these are time-consuming and expensive to run. Oculomotor measures seem more appealing, as they are easily accessible in terms of money and time.

 Recently, the World Federation of Societies of Biological Psychiatry and the World Federation of ADHD have identified the need for dedicated biomarkers of ADHD (2). Indeed, basic oculomotor variability has been proposed as a potential biomarker for ADHD (3; but see 4). However, it is crucial for any biomarker to show intra-individual reliability (5), and the reliability of basic oculomotor variability has not been investigated. The aim of the current paper is therefore twofold. First, we aim to examine whether oculomotor variability during resting-state based paradigms: 1) shows intra-individual reliability, and secondly, 2) correlates with ADHD tendencies (as a whole or either of its two subscales, inattention and hyperactivity), and two commonly associated traits, mind wandering and impulsivity. 

### Oculomotor functioning and variability

While ‘saccades’ refer to sudden, ballistic movements in eye position and ‘fixations’ refer to the maintenance of the eye position on a particular spot, microsaccades refer to small, sudden movements of the eye position during fixations (see (1) for a review). Microsaccades are one of three types of fixational eye movements, the others being drift and tremor. The movements of microsaccades have been described as ‘jerk-like’, small (typically below 1-2° in amplitude), and often as ‘binocular’ (i.e., in both eyes simultaneously). Suggestions on the purposes of microsaccades include control over fixation position, prevention of perceptual fading, improvement of visual processing, (small-area) scanning of the environment, and acuity (see (1, 6) for reviews). 

While microsaccades have been related to attention, this refers mostly to attentional cuing and ‘covert attention’ (i.e., foci of attention separate from the current eye position). Attentional cuing has been known to modulate both the direction and occurrence of microsaccades, with the latter commonly showing the ‘microsaccade rate signature’ – a sudden drop in microsaccades after cue onset, followed by a strong increase right after. Interestingly, this modulation of microsaccade rate seems influenceable by top-down expectations (7). However, the role of attentional cuing relates to task-related variability, not to the manifestation of variability during rest – which can only be related to fluctuations in internal states.

### Oculomotor variability and ADHD symptomatology

Fried et al. (8) examined task-related differences between adults with ADHD (both in an ‘unmedicated’ and ‘medicated’ session) and healthy controls (unmedicated in both sessions). Participants were asked to press a button in response to targets but not to non-targets. While unmedicated, participants with ADHD showed significantly higher microsaccade and blink rates compared to controls, both near stimulus onset and throughout the entire trial. However, these differences were not found in the ‘medicated’ session. No significant between-group differences were found in pupil size mean or variability. Similarly, a separate study compared microsaccades between participants with and without ADHD in a visual go/no-go task with a fixed inter-stimulus interval (9; also see 10). Microsaccade rate prior to target onset was reduced in controls but not in patients. 

Resting-state based approaches can be found in two recent studies. Panagiotidi et al. (3) instructed participants to fixate on cross for 20 seconds over 20 trials. They found a positive association between microsaccade rate and self-assessed ADHD tendencies within a healthy population (r = .35 on 38 participants), but did not investigate pupil size or blink rate. Unsworth et al. (4) conducted a larger-scale study (N = 204), in which healthy participants had to fixate on a point for five continuous minutes. They found a weak correlation between ADHD tendencies and mean pupil size (r = .15), but not between ADHD and the SD of pupil size, blink rate, or SD of gaze variability. Microsaccades were not analysed. However, as they only used classical significance testing (rather than Bayesian statistics), their analyses cannot assess evidence in favour of the null-hypothesis. Furthermore, their study includes a large number of correlations and is vulnerable to Type I errors. 

None of these studies (3, 4, 9, 10) have examined the reliability of their measures. However, this is a crucial step in investigating individual differences, and specifically biomarkers: If oculomotor measures are not consistent within individuals, it is unclear how their associations with questionnaire scores are meaningful. Likewise, any absences of correlations (4) could potentially be explained by a lack of reliability in the measures.

Intra-individual stability of oculomotor variability has been shown previously over different types of tasks, images, and display modalities (11, 12, 13, 14, 15; see Discussion for more details). However, these studies concern the generalisation of oculomotor variability across different conditions/tasks – and cannot inform us about the repeatability of oculomotor variability, nor about the reliability of basic oculomotor behaviour specifically. 

While Panagiotidi et al. (3) did use an ADHD questionnaire with two subscales – Inattention and Impulsivity/Hyperactivity, reflecting the two main subtypes of ADHD – they only analysed the total scores. However, as the correlation between the subscales was only moderate (r = .46), the subscales show sufficient non-shared variance (78.8%) to investigate their separate contributions. Analysing the subscales separately may still reveal potential differences between them, particularly when it is unclear what exact mechanism underlies the correlation. 

Impulsivity is one of the main characteristics of ADHD (16, 17; although some facets of impulsivity may be more important than others). ADHD has also been associated with increased mind wandering both in clinical samples and in healthy participants (4, 18, 19). Possibly, this reflects a decreased ability to maintain top-down focus.

### Current research 

**Figure 1.**
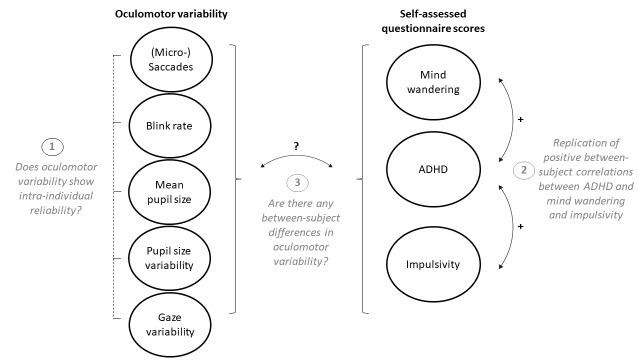
Graphical representation of the oculomotor measures and self-assessed questionnaire scores, with the three aims of the current study.

In the current research, we examine the resting-state paradigm for eye movements in more detail, to see if it produces reliable markers within individuals over different time points (repeatability) and over different conditions (generalisation). In particular, we will examine microsaccade rate, pupil size, blink rate, and gaze variability (in horizontal and vertical dimension). To get further insight into the mechanisms underlying potential individual differences in oculomotor variability, we included self-assessed measures of mind wandering and impulsivity. We aim to replicate positive associations of these two measures with self-assessed ADHD, as well as investigate their relationship to oculomotor variability. Figure 1 shows an overview of our three aims. 

## Methods

### Participants

In total, data of 129 participants was collected. All of them had normal or corrected-to-normal vision. The studies were approved by the local ethics commission.

Experiment 1: Eighty-one participants (66 female, fourteen male, one other, aged between 18-25; exact ages not recorded) contributed in exchange of course credits. Of them, 73 had valid eye tracking data. For three of these remaining 73, the second session was not included because they had more than 33% missing samples.

Experiment 2: Twenty-one participants (eighteen female, 21-40 old, M_age_ = 26.3) contributed in exchange of a monetary reward. All had valid eye-tracking data. Two of them only took part in one test day, due to technical issues. For another three participants, the second session on the first day was excluded, and for one participant, the second session of the second day was excluded, because more than 33% samples were missing.

Experiment 3: Twenty-eight participants (eighteen female, 18-36 years old, M_age_ = 25.5) contributed in exchange of a monetary reward, and twenty-six of them had valid eye tracking data. Of these twenty-six participants, one participant had only three sessions, and another had only two sessions. Furthermore, another eleven (out of 303 remaining) sessions from five different participants were not included because more than 33% missing samples were missing. 

### Design

**Figure 2.**
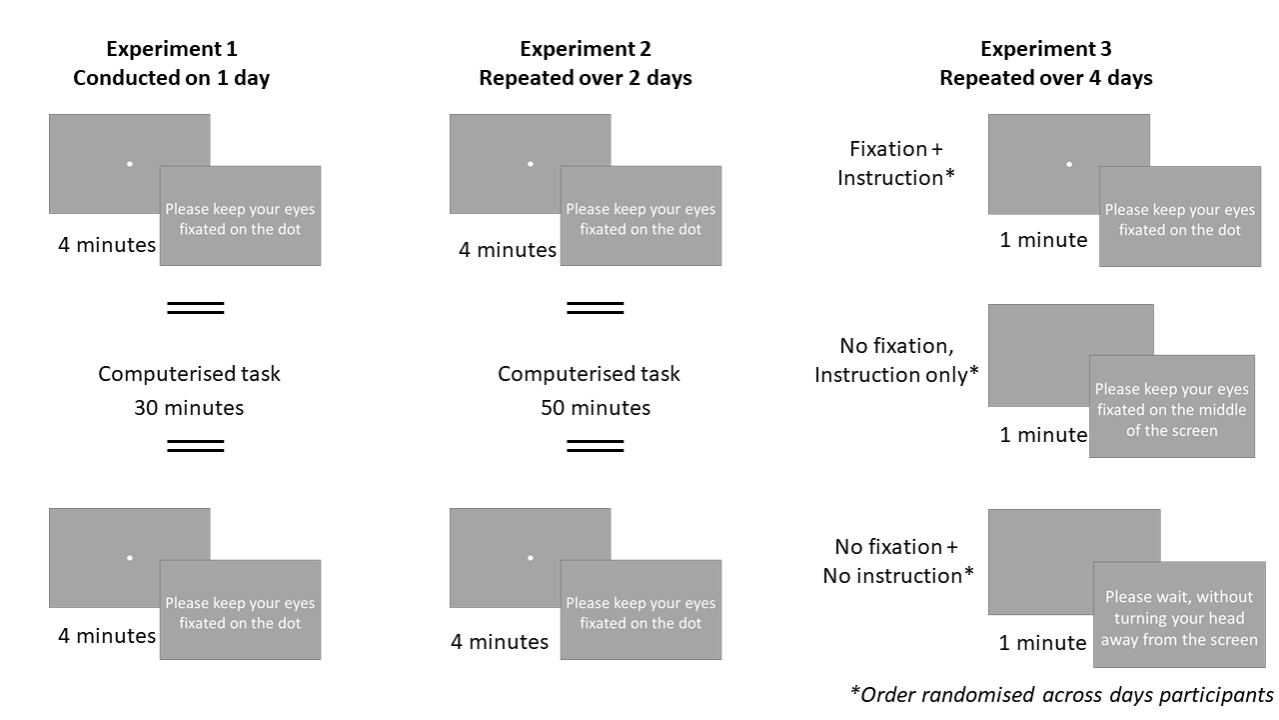
Overview of the resting state eye movement paradigms of all three experiments.

Experiment 1 and 2: Resting state eye movements and pupil dilation were recorded before and after a behavioural task – see Figure 2 for an overview. This gave (2 x 4) 8 minutes of resting state eye measures in total for each participant. ADHD tendencies, mind wandering tendencies, and impulsivity characteristics in daily life were measured with questionnaires. 

Experiment 3. Resting state eye movements and pupil dilation were recorded in three different condition – see Figure 2 for an overview. In the ‘Fixation plus instruction’-condition, participants were asked to fixate on a fixation dot that was displayed on the centre of the screen. In the ‘No fixation, Instruction only’-condition, 

participants were shown a blank screen, and were asked to fixate on the centre of the screen. In the third condition, participants were also shown a blank screen, but were only asked to not turn away from the screen. As they were given no instructions relating to fixation, we refer to the third condition as the ‘No fixation plus no instruction’-condition throughout. This procedure was repeated over four days – resulting in (1 x 3 x 4) 12 minutes of resting state measures for each participant in total. ADHD tendencies, mind wandering tendencies, and impulsivity characteristics in daily life were measured with questionnaires. Again, ADHD tendencies, mind wandering tendencies, and impulsivity characteristics in daily life were measured with questionnaires.

### Materials

The resting state paradigms were generated with MATLAB (The Mathworks, Inc.) and Psychtoolbox-3 (20, 21, 22). The background of the paradigms was set at light-grey, and the fixation point was white. An Eyelink 1000 (SR Research) was used in each of the experiments for eye data recording. Each experiment started calibrating and validating the eye tracker (five-dot calibration in Experiment 1, nine-dot calibration in Experiment 2 and 3). Participants were seated in a chin-rest to limit head movement. 

The Adult ADHD Self-Report Scale (ASRS-v1.1; 23) was administered to measure ADHD tendencies. The ASRS-v1.1 consists of 18 items with a 5-point scale from 0 (“Never”) to 4 (“Very often”) and has a high reliability (with Cronbach's α ranging from .88 to .94; (24, 25)). The ASRS-v1.1 can be divided into two subscales – Inattention and Hyperactivity / impulsivity - reflecting the two main subtypes of ADHD (23, 26).

Furthermore, the Daydreaming Frequency Scale (DFS; 27) was administered to measure mind wandering in daily life. The DFS is a subscale of the Imaginal Processes Inventory and measures the amount of daydreaming and off-task mind wandering in daily life. It consists of 12 items, each with a 5-point scale. It has a high internal consistency (Cronbach's α = .91) and a high test-retest reliability (.76 with an interval of maximum one year; 28).

To measure impulsivity, participants completed the UPPS-P Impulsive Behaviour Scale (29, 30). The UPPS-P consists of 59 items, with a scale ranging from 1 (“agree strongly”) to 4 (“disagree strongly”), divided over five subscales: positive urgency, negative urgency, (lack of) premeditation, (lack of) perseverance, and sensation seeking. 

Experiment 1: The stimuli were generated with a Viglen Genie PC and displayed on an ASUS VG248 monitor with a resolution of 1920 by 1080 and a refresh rate of 144 Hz. Eye movements and pupil dilation were recorded binocularly at 500 Hz. 

Experiment 2: The stimuli were generated on a HP Z230 Workstation PC and an LG 24GM77 monitor with a resolution of 1920 by 1080 and a refresh rate of 120 Hz. The paradigms were displayed on a projector screen. Eye movements and pupil dilation were recorded binocularly at 500 Hz.

Experiment 3: The stimuli were generated with a Bits# Stimulus Processor video-graphic card (Cambridge Research Systems) and a Viglen VIG80S PC, and were displayed on an hp p1230 monitor with a resolution of 1280 by 1024 and a refresh rate of 85Hz. Eye movements and pupil dilation were recorded monocularly at 1000 Hz.

### Procedure

Experiment 1: Participants came to the lab for a session of about 1.5 hours. They were seated at a distance of 615 mm from the screen. Eyes were tracked binocularly during the resting state for four minutes (time 1). Next, participants performed a computerised task, lasting about 30 minutes (data not analysed in the current paper). Right after finishing this task, the resting state paradigm was conducted again (time 2). Lastly, participants filled in nine questionnaires: the DFS, ASRS-v1.1, and UPPS-P, as well as the Beck Anxiety Inventory Second edition (31), Beck Depression Inventory Second edition (32), Short form Wisconsin Schizotypy scales (33), Five-facet Mindfulness Questionnaire (34), Toronto mindfulness scale (35), and Positive and Negative Affect Schedule (36). Only the first three questionnaires were analysed in the current study. 

Experiment 2: Participants came to the lab for two sessions, each about 1.5 hours. They were seated at a distance of 1185 mm to the screen. Eyes were tracked binocularly for four minutes (time 1). Next, they performed a computerised task of about 50 minutes (data not analysed in the current paper), and afterwards they conducted the resting state paradigm again (time 2). Lastly, participants filled in the DFS, ASRS-v1.1, and UPPS-P. 

Experiment 3: The experiment consisted of four sessions of about an hour. Participants were seated at a distance of 1040 mm to the screen. Eyes were tracked monocularly in the three different conditions. Each condition lasted 60 seconds. Instructions were shown for two seconds. For each participant, the order of the conditions was random on each of the four sessions. After completing the resting state eye movements paradigm, participants completed a 30 to 45 minutes computerised task (data not analysed in the current paper). On the last day, they filled in the DFS, ASRS-v1.1, and UPPS-P.

### Procedure

Blinks were defined as missing tracking data, with a maximum of 1000 ms. The total number of blinks throughout each session was counted, and a blink rate per second was subsequently calculated. Pupil size variability was calculated by dividing the standard deviation of the pupil size throughout each session by the mean pupil size – reflecting the coefficient of variation (CV). Gaze variability was calculated separately for the x- and y-screen dimension by calculating the standard deviation of position in degrees throughout the entire session (these standard deviations were not normalised by the mean, as the mean degrees in the middle of the screen is approximately zero). To minimise noise, 20 ms were excluded both before and after missing samples from the calculation of the pupil size mean, pupil size variability, and gaze variability. 

 Microsaccade detection was done with the Engbert and Kliegl algorithm (37), using the Microsaccade Toolbox for R (38). This algorithm calculates a detection threshold from the standard deviation of the velocity distribution multiplied by a value of λ. Whenever the velocity on a sample passes over said threshold in both eyes simultaneously, it is counted as a saccade. It should be noted that the existence of monocular microsaccades remains a controversial topic: Some argue that they are noise, while others have argued they represent more than that (see (39) for an in-depth discussion). For our more practical purposes, we only analysed the well-established binocular microsaccades. Microsaccade-related analyses were therefore only conducted for Experiment 1 and 2, as recordings in Experiment 3 were monocular. 

In accordance with the R toolbox, we report results using a λ value of five for all analyses. As prior research has also used a more stringent λ of 6 (the original Engbert and Kliegl (37), as well as Panagiotidi et al. (3)), we also ran all microsaccade-related analyses with λ = 6 instead. This did not change any of the results patterns. To reduce noise in the detection process, saccades were defined as being at least three samples long. Furthermore, a period of 100 ms both prior and following blinks was excluded. Missing/excluded samples were subsequently interpolated. To avoid the false detection of post-saccadic oscillations as microsaccades, a window of 20 ms following each saccade was excluded. Saccades with amplitudes above 2° or with peak velocities above 200°/s were excluded from subsequent analyses. To validate the microsaccades, saccade amplitude was correlated with velocity over all participants and over both time points (also known as the ‘main sequence’). These were highly correlated with each other for both Experiment 1 (r = .88, BF10 = ∞, p < .001) and for Experiment 2 (r = .86, BF10 = ∞, p < .001). The mean microsaccade rate was 1.1 per second (SD = .43) for Experiment 1 and 1.58 (SD = 47) for Experiment 2, which is within the typical rate of 1-2 per second (40).

Scores on items of the questionnaires were reversed when necessary. Missing responses were substituted with the median (but note that the number of missing responses was negligible, 0.26%). Next, the total score was calculated for each of questionnaire. Individual item scores were used to check the questionnaires’ internal consistency (Cronbach’s α; 41) – see Table 1 for an overview. 

**Table 1:** Overview of the Daydreaming Frequency Scale (DFS), the Adult ADHD Self-Report Scale (ASRS-v1.1), and the UPPS-P Impulsive Behaviour Scale (UPPS-P). Shown are the mean scores and standard deviations (SD) over all the participants, as well as the internal consistency (Cronbach’s α) for each questionnaire, for each sample separately as well as for the combined data. Also shown are the minimum and maximum possible scores of each questionnaire.

Questionnaire	Sample	Mean score	SD	Cronbach’s α	Possible range
DFS	Exp 1	39.3	9.8	.93	
	Exp 2	39.3	8.7	.92	
	Exp 3	37.7	9.1	.93	
	Combined	39.0	9.4	.92	12-60
ASRS-v1.1	Exp 1	33.4	8.5	.81	
	Exp 2	28.5	5.7	.62	
	Exp 3	25.5	7.3	.76	
	Combined	30.6	8.6	.89	0-72
UPPS-P	Exp 1	138.8	23.9	.93	
	Exp 2	119.7	20.3	.93	
	Exp 3	122.8	18.6	.68	
	Combined	132.3	23.7	.92	59-236

All Bayesian statistics throughout the current research were conducted in JASP (42), using the default options of equal prior probabilities for each model and 10000 Monte Carlo simulation iterations. Distributions of the oculomotor measures were highly skewed on the group level. This may bias the results of the correlation analyses, particularly for Experiment 2 and 3, which have smaller sample sizes. For consistency, all analyses were conducted on the natural logarithm of the measures. 

Because of the differences in design, the intra-individual reliability was examined separately for each experiment. The individual differences analyses (Aim 2 and 3) were only conducted on the combined data. 

## Results aim 1. Intra-individual reliability of oculomotor variability measures

### Experiment 1. Reliability over time 

**Figure 3.**
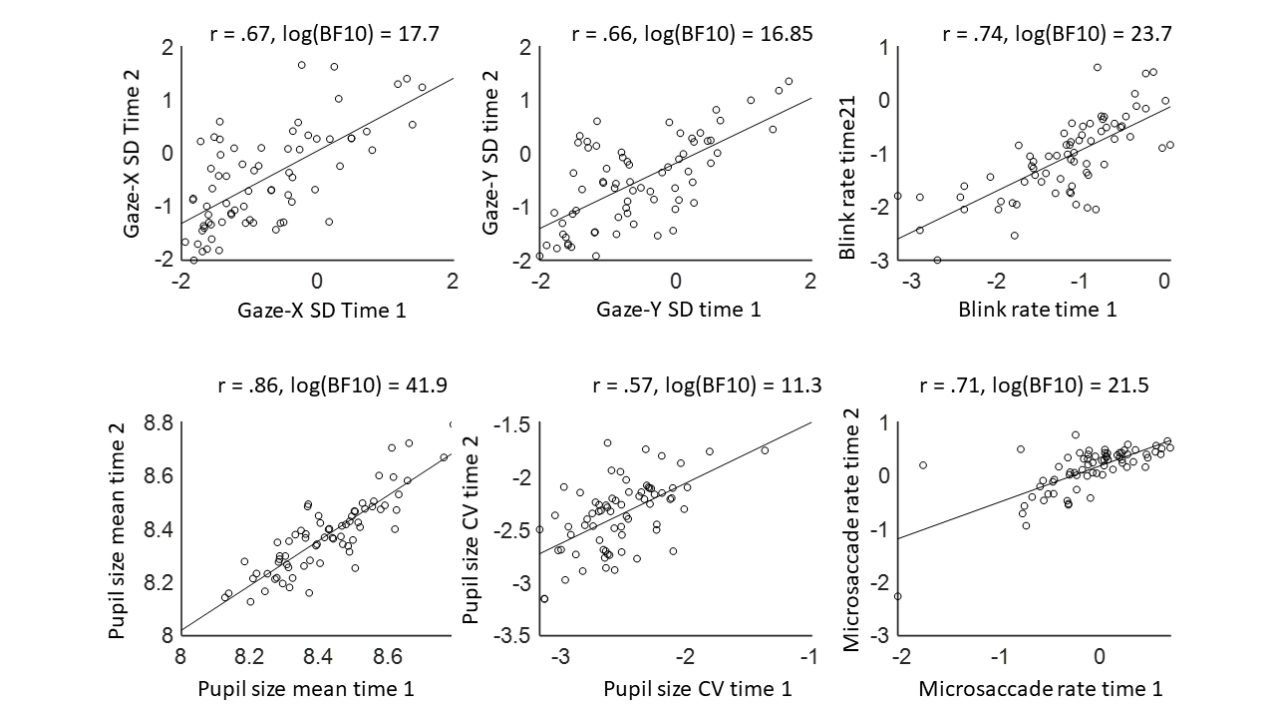
Correlations between time point 1 (pre-task) and time point 2 (post-task) for each of the five oculomotor measures from Experiment 1: Gaze variability (standard deviation; SD) in the horizontal dimension, gaze SD in the vertical dimension, pupil size mean, pupil size coefficient of variability (CV), blink rate per second, and microsaccade rate per second (Ms). All five measures show a high correlation coefficient and accompanying high Bayes Factor, indicating that the measures show intra-individual reliability over time. Note that both the measures and the Bayes Factors are logged.

Two means were calculated for each measure (microsaccade rate, blink rate, pupil size mean, pupil size variability, gaze-x variability, and gaze-y variability): One for time point 1 (pre-task) and one for time point 2 (post-task). Bayesian Pearson pairs were then conducted on each of the measures to test intra-individual reliability over time. Figure 3 shows the within-subject correlational plots over the two time points for the logged measures of gaze variability in the horizontal and vertical dimension, pupil size variability, blink rate, and microsaccade rate – with correlation coefficients and logged Bayes Factors (BF10) on top. 

The BF10 reflect the likelihood of the data for the alternative hypothesis (in this case, the presence of a correlation) over the null-hypothesis (in this case, the absence of a correlation), and can take a value between zero to infinity – note that BF01 (null over alternative hypothesis) can be derived from BF10 (alternative over null) by taking its inverse. To interpret the Bayes Factors, the guidelines from Lee & Wagenmakers (43) were used. It is important to note however that, unlike in classical significance testing, these labels are a heuristic for verbalising results, rather than hard cut offs. For a full interpretation of the Bayes Factor, it is important to look at the ‘raw’ value. For example, for gaze variability in the horizontal dimension, the log(BF10) between time 1 and 2 is 17.7 – meaning that the likelihood of the data is (exp(17.7) = ) 48642102 times larger under the alternative than under the null-hypothesis. This can be interpreted as extremely high evidence for the presence over the absence of a correlation between the two time points. The other four measures show similarly extreme Bayes Factors. Each of the measures show high and positive r-values, indicating that they show intra-individual consistency. Thus, oculomotor shows reliability when measured half an hour apart. 

### Experiment 2. Reliability over time and days

**Figure 4.**
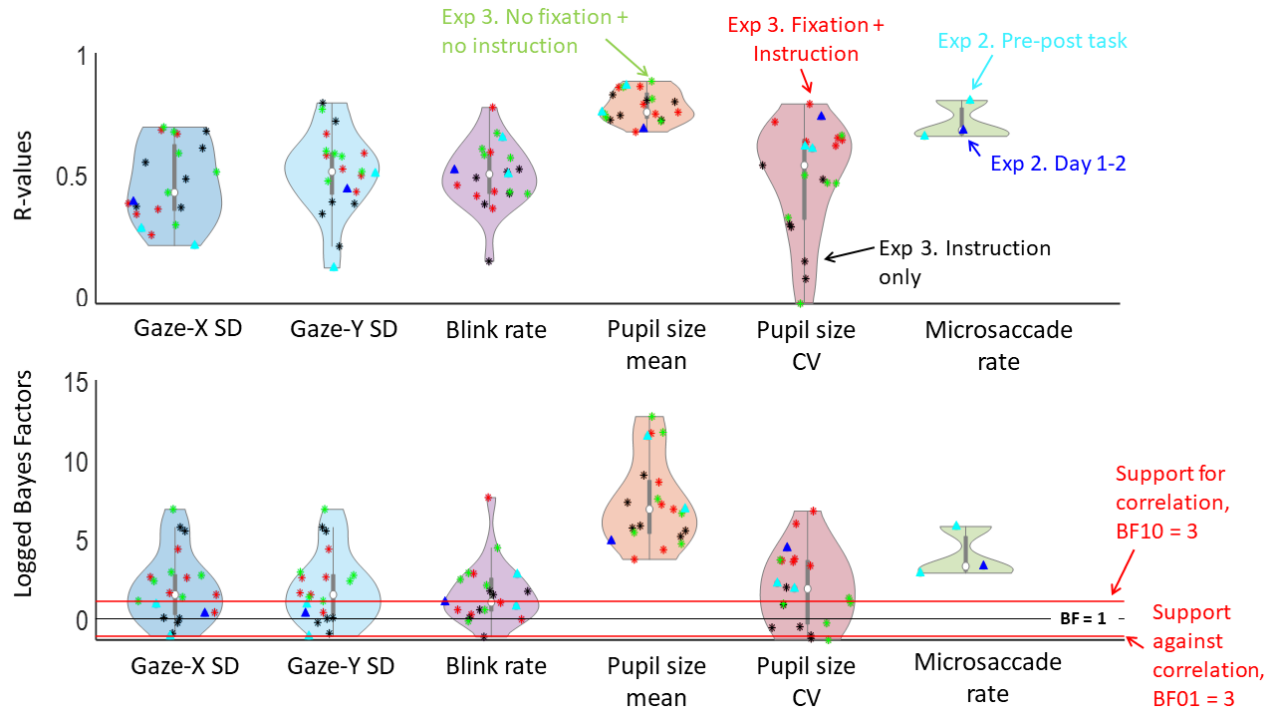
Distributions of the correlation coefficients (top panel) and accompanying logged Bayes Factors (bottom panel) of the correlation analyses on within-subject reliability for each of the five oculomotor measures. Values denoted with a triangle represent the correlations for Experiment 2, with light-blue triangles representing the correlations between different time points (pre and post task), and dark-blue triangles representing the correlation between days. Values denoted with an asterisk represent the correlations for Experiment 3, with red, black, and green representing the different conditions (‘Fixation plus instruction’, ‘No fixation, instruction only’, and ‘No fixation plus no instruction’ respectively). In the top panel, higher values on the y-axis indicate higher correlation coefficients. In the bottom panel, values above the upper red line indicate evidence in favour of the existence of correlations over time, while values below the lower red line (log(BF) < -1) indicate evidence against correlation over time. Values falling between the two red lines are interpreted as indeterminate. Overall, reliability seems low for variability in gaze position, particularly in the horizontal dimension, but the other measures show good reliability.

After we found that the oculomotor markers were reliable within one experimental session, we were tested whether this reliability would hold up over different testing days. Combined, Experiments 2 and 3 have 21 correlation pairs for each oculomotor measure, each testing the reliability over different time points and days. Rather than having to plot each correlation separately and then trying to assess the global patterns, the distributions of these correlations are shown in violin plots (Figure 4). This way of representing the data allows for an immediate overall picture of the correlations. The vertical dimension of these violin plots indicates the entire range of correlation coefficients (top panel) and accompanying Bayes Factors (bottom panel), while the horizontal dimension indicates the density. Each condition is also plotted (coloured triangles and asterisks), with the white dot representing the median value. 

To test the intra-individual reliability over time in Experiment 2, four means were calculated for each measure: One for time 1 (pre-task) and one for time 2 (post-task), both for day 1 and day 2. For both days, Bayesian Pearson pairs were conducted between time 1 and time 2 on each measure – giving two replications of the analysis of Experiment 1 (shown in Figure 4 in light-blue triangles). Again, we found evidence in favour of correlations between time 1 and 2 for pupil size mean and variability, blink rate, and microsaccade rate (with all six BF10 above 1, and only one of them in the indeterminate range), with corresponding r-values all being moderate to high – though mean pupil size is clearly more reliable than the other measures. These findings again indicate good intra-individual reliability of the measures – especially when considering the much smaller sample size of this experiment. These results replicate the findings from Experiment 1 with almost twice as much time in between the two time points. However, we no longer found evidence for intra-individual reliability in gaze variability, especially in the horizontal dimension: All four BF10 were in the indeterminate range, with three of them being below 1. 

Next, means over time points were averaged, resulting in two means for each measure: One for day 1, and one for day 2. Bayesian Pearson pairs were conducted on each of the measures between day 1 and day 2 to test intra-individual reliability on a longer time span. Figure 4 shows the correlation coefficient and Bayes Factor for each measure (dark-blue triangles). The correlations between days show similar patterns to the ones between time points: Gaze variability appears least reliable, while pupil size variability, blink rate, and microsaccade rate show good reliability. 

### Interim-discussion: How long should a resting state session be?

Overall, oculomotor variability showed good intra-individual reliability over time, both before and after a task of 30/50 minutes (Experiment 1 and 2 respectively), as well as over days (Experiment 2) – although variability in gaze position appeared to be the least reliable measure. It should be noted that the differences we found between individuals are substantial – for example, in Experiment 1, for gaze variability in the horizontal dimension at time 1, the most variable participant has an SD that is 32 times larger than the least variable participant. Findings for both experiments were based on a resting state of four minutes. The next question may be how long a resting state session should minimally take before it could be considered to produce reliable measures. To answer this question, we analysed the data of Experiment 1 – looking at variability in gaze and in pupil size over the course of the resting state. 

**Figure 5.**
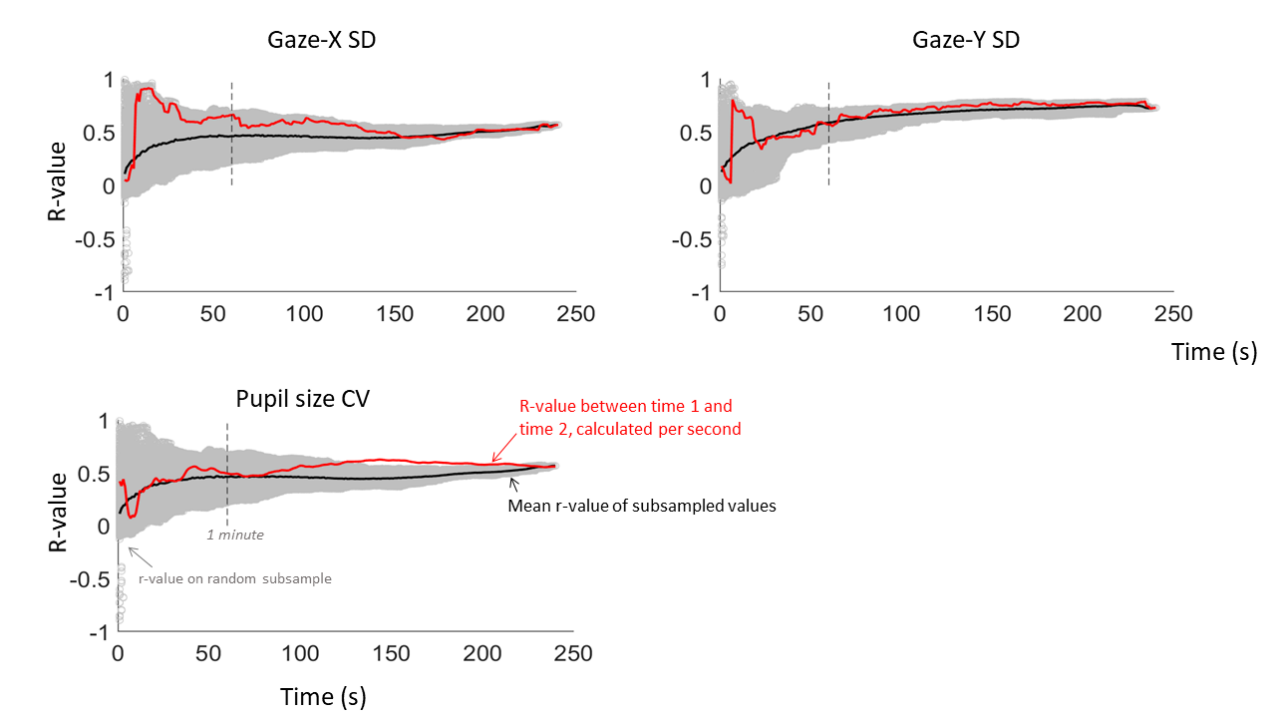
Intra-individual reliability of Experiment 1 over the course of the resting state for our three continuous measures: gaze variability (in the horizontal and vertical dimension) and pupil size mean and variability. The r-value between time points 1 and 2 was calculated at each cumulative second (red), thus reflecting the trajectory over time. Next, for each cumulative second, estimates of the r-value were calculated on 1000 random subsamples. These estimates are shown in light-grey circles, with the mean of these subsamples shown in black.

First, for each measure, the Pearson r-value between time 1 and time 2 was calculated on every cumulative second. This results in 240 r-values – with the first r-value being based on one second of data, and the last r-value being based on four minutes of data. This trajectory reflects how the consistency between the two time points develops as more data is collected (red line on Figure 5). 

Next, we adopted a subsampling approach, using a simplified version of Schönbrodt and Perugini’s (44) approach. From the entire pool of data of four minutes, one chunk of data was randomly selected for both time points, and the r-value between them was calculated. This subsampling was done 1000 times for each cumulative second, represented on Figure 5 by the grey circles, with the mean represented by the black line. This means that, for example, at time = 1 sec, there are 1000 different r-values, each based on one continuous randomly selected second in the entire pool of data. Next, at time = 2 sec, there are also 1000 different r-values, each based on two continuous randomly selected seconds in the data. As such, we end up with 1000 r-values at each cumulative second. Because of this method, the r-values converge to one point as the subsamples are based on more data – resulting in very small margins of error at the right side of the x-axis. Still, the mean trajectory of the subsampled r-values combined with the trajectory of the ‘actual’ r-values can give an idea of the minimal necessary length for an oculomotor resting state. 

Looking at Figure 5, it seems that reliability is lower and more volatile when it is based on less than a minute of data. After one minute, the reliability stabilises, and does not seem to improve any further after two minutes. Based on these outcomes, we recommend that an oculomotor resting state session is no shorter than one minute, but that it may not be necessary to collect more than two minutes of continuous data. However, this conclusion is based solely on the gaze position and pupil size recordings, and not on blink and microsaccade rates (which occur at a much slower time scale). 

### Experiment 3. Reliability over days and conditions

In Experiment 3, we were not only interested in the intra-individual reliability of oculomotor variability over different days (repeatability), but also in the extent to which the oculomotor variability would generalise over different types of ‘oculomotor resting states’. For this, we used the same resting state version as in Experiment 1 and 2, as well as a free viewing version (in which participants did not have to fixate on anything, and were free to look anywhere on the screen), and an ‘intermediate’ version (in which participants were asked to fixate on the middle of the screen, but were not provided with a fixation dot). Because participants were asked to participate in each condition on four different days (resulting in twelve resting states per participant), we made the sessions shorter – using one minute per resting state instead of four. As shown above, this is long enough to produce reliable estimates.

For each of the measures, means were calculated separately for each condition and each day (thus resulting in twelve means for each measure). Bayesian Pearson correlations were conducted for each measure between the means over the different days, separately for each condition (resulting in eighteen correlation pairs for each) – to test the reliability of the oculomotor measures over time. Figure 4 shows these correlation coefficients and Bayes Factors (asterisks) for each of the three conditions (with ‘Fixation plus instruction’ in red, ‘No fixation, instruction only’ in black, and ‘No fixation plus no instruction’ in light-green). The overall pattern is similar to that of Experiment 2. Gaze variability in the horizontal dimension seems least reliable: Bayes Factors mostly show indeterminate evidence against a correlation. Again, mean pupil is clearly the most reliable measure. Pupil size variability and blink rate also performed reasonably well: correlation coefficients for these two measures were mostly moderate to high, with both median values around 0.5. Our ‘intermediate’ condition, in which participants were asked to fixate at the middle of a blank screen, appeared to produce the least reliable measures. 

**Figure 6.**
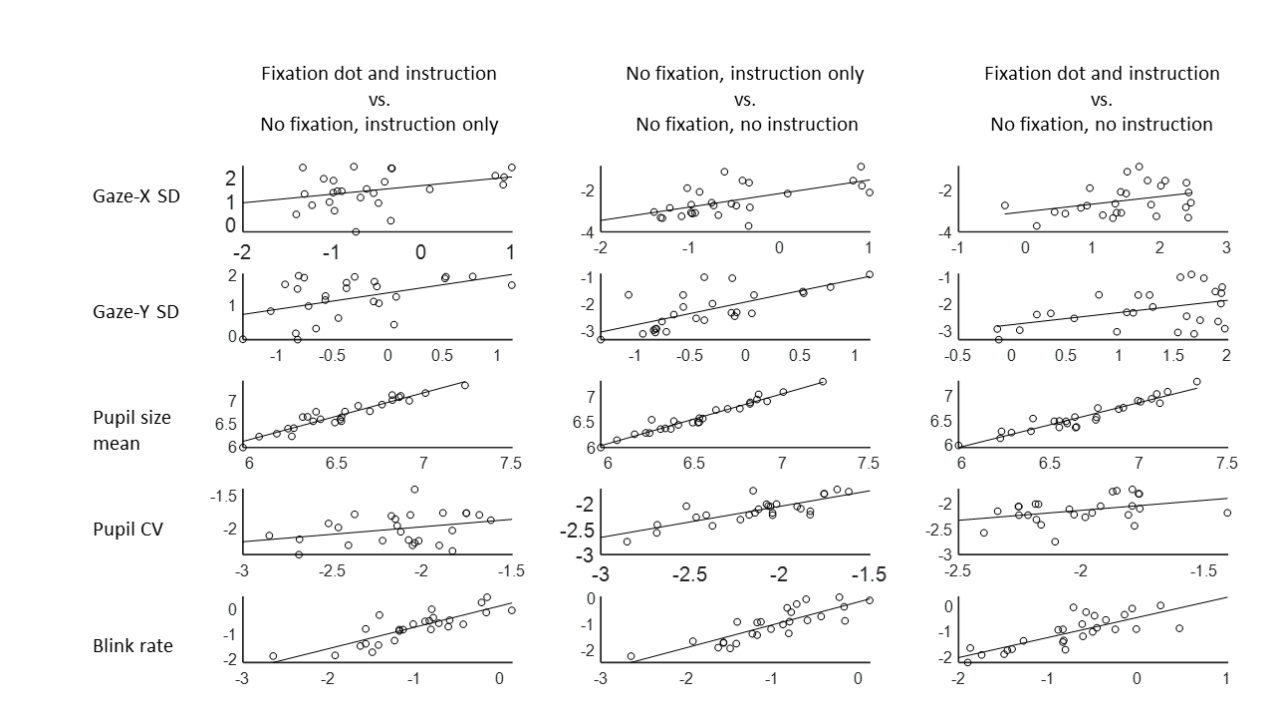
Correlation plots between the three different conditions (‘Fixation plus instruction’, ‘No fixation, instruction only’, and ‘No fixation plus no instruction’) on each of the four oculomotor measures from Experiment 3. Overall, evidence favours the existence of correlations – suggesting good intra-individual reliability of oculomotor variability over the different conditions. Note that the measures are logged.

Over all three experiments, we thus found reliability in oculomotor measures over time, from relatively short ranges (30 to 50 minutes) up to multiple days apart. Next, we were interested in to what extent the oculomotor measures were generalisable over different types of resting states. To examine this, means were averaged over days, resulting in three means for each measure, each reflecting one condition. Bayesian Pearson correlations were conducted on the means of the three conditions – to investigate the reliability of the measures over different conditions. Figure 6 shows the correlation plots between the conditions for each measure, with Table 2 showing the accompanying correlation coefficients and Bayes Factors. All correlations had a Bayes Factor above 1, with eight of them ranging from moderate to extreme. Overall, the measures again show moderate to high reliability, although it is the poorest for gaze variability in the horizontal dimension. Mean pupil size is again the most reliable measure. 

**Table 2:** Overview of the intra-individual reliability across conditions for each of the measures from Experiment 3. For each pair of conditions and each measure, the correlation coefficient is shown, with the accompanying BF10 in brackets.

Measure	Fixation + Instruction vs Instruction only	Fixation + Instruction vs No fixation + Instruction	Instruction only vs No fixation + Instruction
Gaze-X	.36 (1.12)	.63 (60.05)	.37 (1.28)
Gaze-Y	.47 (3.66)	.73 (1241.77)	.45 (3.10)
Pupil mean	.95 (6.1e+10)	.98 (1.3e+14)	.95 (6.4e+10)
Pupil CV	.40 (1.67)	.83 (77689)	.43 (2.41)
Blink rate	.84 (241807)	.85 (288824)	.79 (10224)

Intra-class correlation: The intra-class correlation can estimate the reliability of a larger group of measures, to reflect to what extent they measure the same underlying phenomenon – and as such, can reflect the ‘correlation’ between more than two measures. To estimate the intra-class correlation, a two-way random model was conducted on each measure. The measure of consistency was estimated, as this is most similar to our Pearson correlation analyses. Table 3 shows the correlation coefficients for the average measure, to reflect the overall consistency of the resting states. The analysis was run both on each condition separately as well, to get an estimate of reliability over days, and collapsed over conditions and days, to get an estimate of the overall reliability of the paradigm. 

**Table 3:** Overview of the intra-class correlation coefficients of the average measure for each of the three conditions from Experiment 3, separately for each of the four measures, as well as the coefficients per measure over all conditions and days combined.

Measure	Fixation + Instruction	Instruction only	No fixation + Instruction	All
Gaze-X SD	.74	.77	.83	.85
Gaze-Y SD	.75	.74	.85	.87
Pupil size mean	.91	.90	.92	.97
Pupil size CV	.88	.65	.76	.88
Blink rate	.80	.65	.81	.91

All three conditions showed moderate (.5-.75) to good (.75-.9) reliability (see (45) for guidelines), although results again indicate that the ‘Instruction only’ condition produces the least reliable results. When collapsing over all days and all conditions, reliability is even higher, ranging from good to excellent (.9-1) – though mean pupil size is the only measure that has excellent reliability throughout. Overall, the conditions seem to measure the same underlying construct – reflecting good intra-individual reliability of oculomotor measures. Interestingly, the coefficients are all at least in the good range, even variability in gaze position – as such, diverging from the results of the individual Pearson correlations. However, the Pearson correlations can only reflect the consistency between two single measures, while our intra-class correlations reflect the consistency over all the different days averaged together. This suggests that over all the days combined, the oculomotor variability still shows within-subject consistency. 

## Results aim 2. Between-subject corre-lations between ADHD, mind wander-ing, and impulsivity

Bayesian Person correlations were conducted on the questionnaire scores. Figure 6 shows the between-subject correlational plots with their corresponding Pearson r coefficients and Bayes Factors. Looking at the between-subject correlations between ADHD tendencies, mind wandering (DFS), and impulsivity (UPPS-P), we found that ADHD tendencies were highly correlated with impulsivity and mind wandering tendencies. Both of these findings thus provide extreme evidence for replication of previous literature.

There was also some evidence for a correlation between mind wandering and impulsivity, but the evidence was in a much lower range and the accompanying correlation coefficient was similarly low, Pearson r = .23, BF10 = 3.8. It seems plausible that this correlation is caused by a confounding effect of ADHD tendencies. To statistically control for ADHD tendencies, a Bayesian Linear Regression was performed in which impulsivity scores were regressed on mind wandering tendencies (alternative Model M1) and compared to a null-model that included the ADHD tendencies as model term (model M0; see (46) for more details on this method). Bayesian evidence favoured M0 over M1, BF01 = 7.7, indicating that the relationship between impulsivity and mind wandering disappears when controlling for ADHD tendencies. 

## Results aim 3. No between-subject correlations between questionnaires and oculomotor behaviour

One overall mean was calculated for every participant, separately for each oculomotor measure, collapsed over all time points and conditions. Because the distributions of mean pupil size differed across the three experiments (caused by differences in distance-to-screen, room lighting, et cetera), the values were re-centered separatedly for each experiment (e.g., the mean of pupil size in Experiment 1 was substracted from each individual value in Experiment 1), so they could be combined into one analysis. 

Out of the eighteen analyses, thirteen showed moderate evidence against a correlation, and five were in the indeterminate range (three of them with BF10 < 1, and the other two with BF10 > 1). Looking at the two correlations that had a BF10 > 1 (though in the indeterminate range), the accompanying r-values were low (explaining only 4.4 and 4.8% of the total variance).

To examine if any correlations would be more pronounced when looking at the subscales instead of the total scores of ADHD, the inattention and impulsivity/hyperactivity scores were correlated with the oculomotor measures. Pupil size variability correlated with the inattention subscale (r = .24, BF10 = 3.75), but not with impulsivity/hyperactivity (r = .13, BF10 = .31) – indicating that participants with more inattention-related ADHD tendencies showed more variability in pupil size. However, the explained variance was again low (5.8%). 

**Figure 7.**
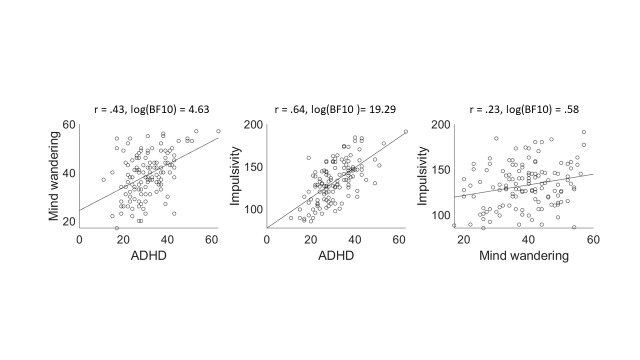
Correlational plots between self-assessed ADHD tendencies, mind wandering tendencies, and impulsivity, with accompanying Pearson r and Bayes Factor values. ADHD tendencies are positively correlated with both mind wandering and impulsivity – replicating previous literature.

**Figure 8.**
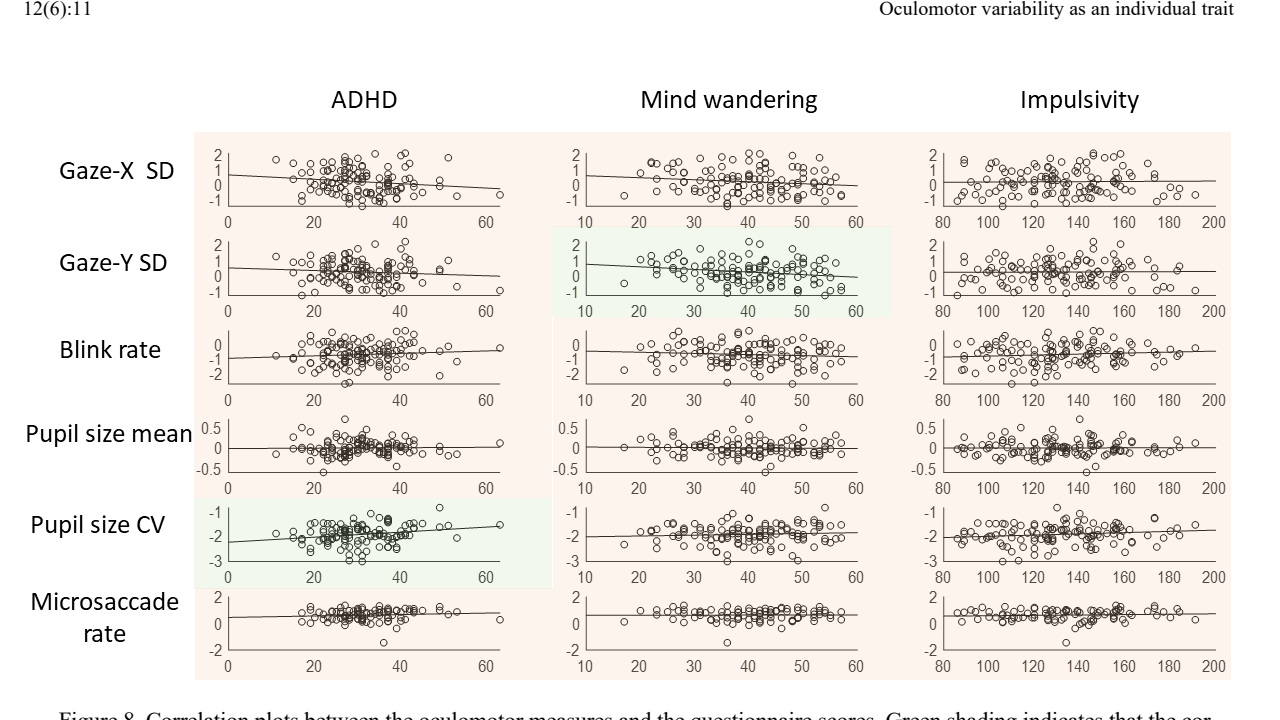
Correlation plots between the oculomotor measures and the questionnaire scores. Green shading indicates that the corresponding Bayes Factor is above 1 (indicating evidence in favour of a correlation between the conditions on that measure), while red shading indicates a Bayes Factor below 1 (indicating evidence against a correlation). Note that the oculomotor measures are logged.

**Table 4:** Kendall’s τ-values (BF10) between the three questionnaires and the measures of oculomotor variability, combined over the three experiments.

Measure	ADHD	Mind wandering	Impulsivity
Gaze-X SD	-.15 (.41)	-.15 (.38)	.02 (.12)
Gaze-Y SD	-.10 (.20)	-.21 (1.61)	.02 (.12)
Pupil size mean	.02 (.12)	-.03 (.12)	.01 (.12)
Pupil size CV	.22 (2.11)	.08 (.16)	.15 (.43)
Blink rate	.11 (.24)	-.09 (.18)	.12 (.27)
Microsaccade rate	.10 (.21)	-.02 (.13)	.08 (.17)

## Discussion

In the current study, we found that oculomotor variability indeed shows consistency within individuals, both over time (repeatability) and over different conditions (generalisation). Of the six measures that we used (variability in both horizontal and vertical dimensions, pupil size mean and variability, blink rate, and microsaccade rate), each showed consistency to some extent - though, mean pupil size was the only measure that showed excellent reliability throughout all the analyses. Notably, microsaccade rate also appeared to have great reliability, but as we did could not extract these in Experiment 3, more research is needed on the generalisation of this marker. Furthermore, we mostly found evidence against correlations, and for the few correlations that were weakly supported, effects sizes were low – mirroring Unsworth et al. (4). We did find positive correlations between self-assessed traits, replicating previous associations between ADHD and mind wandering (18, 19), and between ADHD and impulsivity (16, 17).

### Reliability of oculomotor variability 

To our knowledge, the present study is the first systematic investigation of the repeatability of standard measures of oculomotor activity. Intra-individual reliability of oculomotor variability has previously been investigated in the context of generalisation across different tasks (11, 12, 13, 14, 15). In particular, Andrews and Coppola (11) looked at fixation duration and saccade size across five conditions: a ‘dark room’ condition, in which participants’ basic oculomotor behaviour was continuously recorded for 100 seconds, two free viewing conditions (simple and complex patterns), and two ‘cognitive’ tasks (visual search and reading). Basic oculomotor measures showed positive intra-individual correlations with the viewing conditions, but not with cognitive conditions. Similarly, Poynter et al. (14) extracted six measures of oculomotor activity (saccade amplitude, microsaccade rate and amplitude, and fixation rate, duration, and size – the last one being a measure of all three fixational eye movements combined) over four different tasks (a sustained fixation, scan-identify, search, and Stroop task), and found that each oculomotor measure was reliable across tasks. However, their fixation-task trials were only three seconds long – meaning that activity is highly dependent on stimulus-onset. While these studies have compared measures across tasks, they did not investigate how repeatable these measures are within individuals. Instead, our results show that participants who show high variability in one session tend to show high variability in all sessions. This is an important condition for studying individual traits. 

Previous studies have found similar intra-individual reliabilities in reaction time variability over time within and across tasks (47, 48, 49; but see 50). In these contexts, it is difficult to quantify which part of the variability is task-related or task-unrelated. It is possible that found consistencies reflect individual consistency in viewing and processing strategies. To our knowledge, our design is the first to investigate the intra-individual stability in variability in basic oculomotor behaviour using continuous measurement under an absence of changes in the external environment. 

Reliability over time was strongest in Experiment 1 – in which the two measures were closest together in time – and lowest in Experiment 3 – in which measures were typically separated by multiple days. Still, the measures showed at least moderate intra-individual consistency even in Experiment 3. Of course, the individual correlation pairs will be affected by chance. This is evidenced by the distribution plots in Figure 5, that shows a large range of correlation coefficients. Still, the overall distributions favoured moderate to high correlations, with median r-values around .5 (with the exception of gaze variability). Furthermore, intra-class correlation coefficients showed good to excellent consistency for each of the measures over days – revealing that they likely reflect the same underlying construct. 

Gaze variability was consistently the weakest measure, particularly in the horizontal dimension. In the current analyses, the gaze-position over the horizontal and vertical dimensions were examined separately. Another measure to quantify fixation stability used in the literature is the ‘Bivariate Contour Ellipse Area’ (BCEA; 51), representing the area in which P % of fixations occur. We calculated the BCEA with P = 68%, and reran the analyses on these values. Reliability of the BCEA was comparable to the reliability of gaze variability in the vertical plane only. It should be noticed that there was evidence for a correlation between BCEA and ADHD tendencies, though the direction was negative (r = -.30, BF10 = 23.1). Remarkably, when examining the BCEA distribution, we noticed the values have an extremely large range between participants (minimum value = 234, maximum value = 457257). Visual inspection of the data revealed the larger BCEA values appeared to be driven by partial blinks – i.e., sudden large jumps in eye position, without complete loss of signal – suggesting that participants with more ADHD tendencies made less partial blinks. The meaning and significance of this remains open to interpretation. 

One possibility for the weaker performance of gaze variability compared to the other measures is that it is driven by a multitude of sources, including saccades, drift, tremor, and partial blinks. Gaze variability may have less specificity than the other measures, and thus, less validity. While reliability and validity are theoretically different constructs, in practice, they often go hand in hand. While simple gaze position has computational appeal, more specified measures could be more informative of underlying constructs.

It is important to note that we find oculomotor behaviour is consistent within individuals over time – likely reflecting individual traits. This means that individuals who are highly variable at time 1 typically are also highly variable at time 2. However, this does not mean that the measures are exactly the same at time 1 and time 2; they are still subject to variability. 

### Statistical power and sample size 

Despite our relatively large sample size, a number of between-subject analyses in Aim 3 produced indeterminate Bayes Factors. If anything, this highlights the importance of large samples when studying individual differences. However, sample size is not the only determinant of statistical power (52, 53). Among others, one can obtain higher power by minimising measurement noise and collecting enough data points with reliable measurements. Our results diverge from previous literature, which found a positive association between ADHD and microsaccade rate in a healthy population (3). We used the same eye tracker system, refresh rate, microsaccade detection algorithm (37), and analysis (Pearson’s r). However, our study had a higher sample size (our correlation between ADHD and microsaccades included 94 participants, compared to their 38) and more data points (minimally 8 compared to ~6.5 minutes). The absence of a replication in our results is thus not caused by a lack of power. 

### Individual differences in oculomotor variability 

The current between-subject analyses replicate Unsworth et al. (4), who tested over 200 participants (though they did not analyse microsaccades) – and found that inter-individual correlates of oculomotor measures are not robust and typically insignicant. With our Bayesian analyses, we furthermore show explicit evidence against the individual differences. 

In our experiments, oculomotor variability was recorded in one continuous ‘trial’, while in Panagiotidi et al. (3) participants fixated for only 20 seconds in a row over 20 separate trials. After each trial, they were given a break, and could decide themselves when to continue. One possibility is that the observed relationship with ADHD is driven by reduced ability to switch between trials and breaks, related to deficits in executive functioning (see (54) for a meta-analysis). For now, this remains speculative, as the break-to-task switch times were not investigated. 

Our results also diverge from Fried et al. (8) and Danker et al. (9), but there are profound differences in design: Their participants performed a rapid action selection task with trials of 2 seconds long, that featured a visual stimulus in each trial – and as such, capture functional, task-related variability. Microsaccades likely do not differ in ADHD patients per se, but rather may be task-dependent (e.g., (55), in which both microsaccade rates and performance in cued visual orientation discrimination tasks was not significantly different between ADHD patients and healthy controls). As such, our results on ADHD and microsaccades are in line with Roberts et al. (52).

 Our sample did not include many individuals at the high end of the spectrum, possibly restricting our effect sizes. In healthy and academic samples, these more extreme cases will be difficult to find by chance, particularly in small samples. More definitive conclusions would require larger sample sizes, or oversampling for extreme scores. Last, oculomotor measures may still prove useful to distinguish clinical (or extreme) cases of ADHD, further characterise the dysfunctional circuitry underlying the disorder or assess the possible benefits of medication. 

### Variability during rest: beneficial or detrimental? 

Within the context of our study, we have discussed possible associations between oculomotor variability and ADHD. This may imply that oculomotor variability is inherently detrimental. Of course, this would be a false assumption; oculomotor variability inherently reflects the functioning of our oculomotor system. Fixational eye movements have been proven to be important for our vision (see (1, 6) for reviews), and more generally speaking it is possible that intra-individual variability is largely irreducible (56).

When participants are instructed to keep fixation, higher variability may be perceived as ‘worse performance’. On the other hand, because fixational eye movements are a healthy phenomenon during fixation, it is therefore unclear whether we should expect them to be reduced or increased in clinical conditions. This highlights the importance of indicating which mechanisms would drive potential individual differences in variability. Instead, task-based oculomotor variability, in which certain eye movement patterns may be considered as beneficial or detrimental for the task, may be better suited to study these individual differences.

### Oculomotor measures: extraction and correlations 

In the current analyses, we only included saccades with an amplitude below two degrees in the microsaccade rate, similar to (3, 8). Although this cut-off is a traditional standard in the literature, it remains somewhat arbitrary. Saccades and microsaccades may represent a continuum, rather than two opposing categories (57, 58). We therefore reran our (micro-) saccades analyses without an amplitude cut-off, to capture more of participants’ total variability. This did not change any of our findings.

We likewise used a cut-off for the blink extraction: Blinks were computed as missing samples with a maximum of one second – to differentiate blinks from periods of task disengagement (e.g., a participant falling asleep). Similarly, when rerunning our blink-related analyses without the upper-bound cut-off, our findings did not change. 

To extract the microsaccades, we used the binocular detection algorithm of Engbert and Kliegl (37). One feature of this algorithm is that the microsaccade detection threshold is computed for each trial, to adjust for different noise levels across different trials. However, our tasks do not contain any traditional trials, only continuous measurements. This may affect the computation detection threshold due to untypical variability within the ‘trial’, resulting in too lenient thresholds. Still, our microsaccade rate is well in line with previously reported rates using shorter trials. Furthermore, we also used the measures of gaze variability, which may capture both and other types of fixational eye movements – thus reflecting an overall capacity to fixate. 

Previous research has also looked at the associations between task-based oculomotor measures, and found that different measures (saccade amplitude, microsaccade rate and amplitude, and fixation rate, duration, and size) could all be captured by one single factor (14) – which they interpret as “Individuals’ eye-movement behavior profiles”. Follow-up analyses show this was not the case in our data: Only three out of nineteen pairs of measures showed clear evidence for a correlation. Two indicated low correlations between pupil size variability and microsaccade and blink rate (r = .31 and .24 respectively), while the last one was an unsurprising high correlation between the horizontal and vertical dimension of gaze variability (r = .82). However, our measures are quite different from Poynter et al. (14), with only microsaccade rate overlapping (see Reliability of oculomotor variability Section). 

### Conclusion 

In the current study, we found that oculomotor variability shows good correlation within individuals both over time and over different conditions. Particularly mean pupil size had very high reliability. Still, microsaccade rate, blink rate, and variability of pupil diameter show reasonable reliability – meaning that these measures may have the potential to be used as biomarkers. Of course, this begs the question of what for they can be used as biomarkers. Our results showed that the between-subject correlations to self-assessed ADHD, mind wandering, and impulsivity were all either absent or very small. In contrast, the questionnaires themselves correlated well with each. Still, it is possible that these oculomotor measures may serve a function complementing questionnaires or show stronger validity, for instance in predicting important outcomes. Future research should focus on linking the resting-state oculomotor measures to task-related deficiencies in ADHD or differences in brain structure or integrity, as in these cases, oculomotor measures may serve as an easy and cheap substitute.

## Ethics and Conflict of Interest

The author(s) declare(s) that the contents of the article are in agreement with the ethics described in http://biblio.unibe.ch/portale/elibrary/BOP/jemr/ethics.html and that there is no conflict of interest regarding the publication of this paper. 

## Acknowledgements

We would like to thank Rachel Draper, Laura Daniells, Laura Fleetwood, and Joel Bentley, who collected the data for Experiment 1, as well as Lingsi Zhou, who helped collect the data for Experiment 2, and Natasha Harris, Humairaa Uddin, and Sarah Lethbridge, who helped collecting the data for Experiment 3. We are grateful to Engbert et al. for sharing their code online and to Sam Hutton, for his helpful advice on microsaccade detection and for his code for excluding post-saccadic oscillation. 

## References

[b25] Adler, L. A. , Spencer, T. , Faraone, S. V. , Kessler, R. C. , Howes, M. J. , Biederman, J. , & Secnik, K. ( 2006). Validity of pilot Adult ADHD Self- Report Scale (ASRS) to Rate Adult ADHD symptoms. Annals of Clinical Psychiatry, 18( 3), 145–148. 10.1080/10401230600801077 1040-1237 16923651

[b24] Adler, L. A. , Shaw, D. M. , Spencer, T. J. , Newcorn, J. H. , Hammerness, P. , Sitt, D. J. , Minerly, C. , Davidow, J. V. , & Faraone, S. V. ( 2012). Preliminary examination of the reliability and concurrent validity of the attention-deficit/hyperactivity disorder self-report scale v1.1 symptom checklist to rate symptoms of attention-deficit/hyperactivity disorder in adolescents. Journal of Child and Adolescent Psychopharmacology, 22( 3), 238–244. 10.1089/cap.2011.0062 1044-5463 22537184

[b11] Andrews, T. J. , & Coppola, D. M. ( 1999). Idiosyncratic characteristics of saccadic eye movements when viewing different visual environments. Vision Research, 39( 17), 2947–2953. 10.1016/S0042-6989(99)00019-X 0042-6989 10492820

[b52] Asendorpf, J. B. , Conner, M. , Fruyt, F. D. , Houwer, J. D. , Denissen, J. J. A. , Fiedler, K. , . . .. ( 2013). Recommendations for increasing replicability in psychology. European Journal of Personality, 27( 2), 108–119. 10.1002/per.1919 0890-2070

[b34] Baer, R. A. , Smith, G. T. , Hopkins, J. , Krietemeyer, J. , & Toney, L. ( 2006). Using self-report assessment methods to explore facets of mindfulness. Assessment, 13( 1), 27–45. 10.1177/1073191105283504 1073-1911 16443717

[b31] Beck, A. T. , & Steer, R. (1993).The Beck Anxiety Inventory.The Psychological Corporation.

[b32] Beck, A. T. , Steer, R. , & Brown, G. K. (1996).The Beck Depression Inventory-II.Psychological Corporation.

[b16] Berg, J. M. , Latzman, R. D. , Bliwise, N. G. , & Lilienfeld,S. O. (2015).Parsing the heterogeneity of impulsivity: A meta-analytic review of the behavioral implications of the UPPS for psychopathology. Psychological Assessment,27(4),1129–1146. 10.1037/pas0000111 1040-3590 25822833

[b12] Boot,W. R. , Becic,E. , & Kramer,A. F. (2009).Stable individual differences in search strategy? The effect of task demands and motivational factors on scanning strategy in visual search. Journal of Vision (Charlottesville, Va.),9(3),7.1–16. 10.1167/9.3.7 1534-7362 19757946

[b20] Brainard,D. H. (1997).The Psychophysics Toolbox. Spatial Vision,10(4),433–436. 10.1163/156856897X00357 0169-1015 9176952

[b13] Castelhano,M. S. , & Henderson,J. M. (2008).Stable individual differences across images in human saccadic eye movements. Canadian Journal of Experimental Psychology,62(1),1–14. 10.1037/1196-1961.62.1.1 1196-1961 18473624

[b40] Ciuffreda,K. J. , & Tannen,B. (1995).Eye movement basics for the clinician.Mosby.

[b41] Cronbach LJ .(1951). Coefficient alpha and the internal structure of tests. Psychometrika, 16(3),297–334.9.

[b9] Dankner,Y. , Shalev,L. , Carrasco,M. , & Yuval-Greenberg,S. (2017).Prestimulus Inhibition of Saccades in Adults With and Without Attention-Deficit/Hyperactivity Disorder as an Index of Temporal Expectations. Psychological Science,28(7),835–850. 10.1177/0956797617694863 0956-7976 28520552

[b37] Engbert,R. , & Kliegl,R. (2003).Microsaccades uncover the orientation of covert attention. Vision Research,43(9),1035–1045. 10.1016/S0042-6989(03)00084-1 0042-6989 12676246

[b38] Engbert R , Sinn P , Mergenthaler K , Trukenbrod H. (2015). Microsaccade Toolbox.

[b8] Fried,M. , Tsitsiashvili,E. , Bonneh,Y. S. , Sterkin,A. , Wygnanski-Jaffe,T. , Epstein,T. , & Polat,U. (2014).ADHD subjects fail to suppress eye blinks and microsaccades while anticipating visual stimuli but recover with medication. Vision Research,101,62–72. 10.1016/j.visres.2014.05.004 0042-6989 24863585

[b28] Giambra,L. M. (1979-1980).Sex differences in daydreaming and related mental activity from the late teens to the early nineties. International Journal of Aging & Human Development,10(1),1–34. 10.2190/01BD-RFNE-W34G-9ECA 0091-4150 478659

[b47] Hultsch DF , MacDonald SWS , Dixon RA .(2002). Variability in reaction time performance of younger and older adults. The Journals of Gerontology: Series B, 57(2),101–115. 10.1093/geronb/57.2.P101 11867658

[b42] JASP Team. JASP (Version 0.8.5). 2017.

[b23] Kessler,R. C. , Adler,L. , Ames,M. , Demler,O. , Faraone,S. , Hiripi,E. , Howes,M. J. , Jin,R. , Secnik,K. , Spencer,T. , Ustun,T. B. , & Walters,E. E. (2005).The World Health Organization Adult ADHD Self-Report Scale (ASRS): A short screening scale for use in the general population. Psychological Medicine,35(2),245–256. 10.1017/S0033291704002892 0033-2917 15841682

[b21] Kleiner,M. , Brainard,D. H. , & Pelli,D. (2007).What’s new in Psychtoolbox-3? Perception,36(14),1–16.0301-0066

[b45] Koo,T. K. , & Li,M. Y. (2016).A guideline of selecting and reporting intraclass correlation coefficients for reliability research. Journal of Chiropractic Medicine,15(2),155–163. 10.1016/j.jcm.2016.02.012 1556-3707 27330520 PMC4913118

[b35] Lau,M. A. , Bishop,S. R. , Segal,Z. V. , Buis,T. , Anderson,N. D. , Carlson,L. , Shapiro,S. , Carmody,J. , Abbey,S. , & Devins,G. (2006).The Toronto Mindfulness Scale: Development and validation. Journal of Clinical Psychology,62(12),1445–1467. 10.1002/jclp.20326 0021-9762 17019673

[b43] Lee MD , Wagenmakers EJ (2013).Bayesian data analysis for cognitive science: A practical course.

[b29] Lynam,D. R. , Smith,G. T. , Whiteside,S. P. , & Cyders,M. A. (2006).The UPPS-P: Assessing five personality pathways to impulsive behavior (Technical Report).Purdue University.

[b6] Martinez-Conde,S. , Otero-Millan,J. , & Macknik,S. L. (2013).The impact of microsaccades on vision: Towards a unified theory of saccadic function. Nature Reviews. Neuroscience,14(2),83–96. 10.1038/nrn3405 1471-003X 23329159

[b5] Mayeux,R. (2004).Biomarkers: Potential uses and limitations. NeuroRx,1(2),182– 188. 10.1602/neurorx.1.2.182 1545-5343 15717018 PMC534923

[b53] McClelland,G. H. (2000).Increasing statistical power without increasing sample size. The American Psychologist,55(8),963–964. 10.1037/0003-066X.55.8.963 0003-066X

[b10] Mihali,A. , Young,A. G. , Adler,L. A. , Halassa,M. , & Ma,W. J. T. (2018).Perceptual and executive behavioral deficits in ADHD and their differential correlation with microsaccade rate. Biological Psychiatry,83,S200. 10.1016/j.biopsych.2018.02.523 0006-3223

[b17] Miller,D. J. , Derefinko,K. J. , Lynam,D. R. , Milich,R. , & Fillmore,M. T. (2010).Impulsivity and Attention Deficit-Hyperactivity Disorder: Subtype Classification Using the UPPS Impulsive Behavior Scale. Journal of Psychopathology and Behavioral Assessment,32(3),323–332. 10.1007/s10862-009-9155-z 0882-2689 21765593 PMC3137261

[b39] Nyström,M. , Andersson,R. , Niehorster,D. C. , & Hooge,I. (2017).Searching for monocular microsaccades - A red Hering of modern eye trackers? Vision Research,140,44–54. 10.1016/j.visres.2017.07.012 0042-6989 28822717

[b58] Otero-Millan,J. , Troncoso,X. G. , Macknik,S. L. , Serrano-Pedraza,I. , & Martinez-Conde,S. (2008).Saccades and microsaccades during visual fixation, exploration, and search: Foundations for a common saccadic generator. Journal of Vision (Charlottesville, Va.),8(14),21.1–18. 10.1167/8.14.21 1534-7362 19146322

[b57] Otero-Millan,J. , Macknik,S. L. , Langston,R. E. , & Martinez-Conde,S. (2013).An oculomotor continuum from exploration to fixation. Proceedings of the National Academy of Sciences of the United States of America,110(15),6175–6180. 10.1073/pnas.1222715110 0027-8424 23533278 PMC3625326

[b3] Panagiotidi,M. , Overton,P. , & Stafford,T. (2017).Increased microsaccade rate in individuals with ADHD traits. Journal of Eye Movement Research,10(1).1995-8692 10.16910/jemr.10.1.6 PMC7141051 33828642

[b22] Pelli,D. G. (1997).The VideoToolbox software for visual psychophysics: Transforming numbers into movies. Spatial Vision,10(4),437–442. 10.1163/156856897X00366 0169-1015 9176953

[b56] Perquin,M. N. , Yang,J. , Teufel,C. , Sumner,P. , Hedge,C. , & Bompas,A. (2019).Inability to improve performance with control shows limited access to inner states. Journal of Experimental Psychology. General. 0096-3445 31380694 10.1037/xge0000641

[b14] Poynter,W. , Barber,M. , Inman,J. , & Wiggins,C. (2013).Individuals exhibit idiosyncratic eye-movement behavior profiles across tasks. Vision Research,89,32–38. 10.1016/j.visres.2013.07.002 0042-6989 23867568

[b15] Rayner,K. , Li,X. , Williams,C. C. , Cave,K. R. , & Well,A. D. (2007).Eye movements during information processing tasks: Individual differences and cultural effects. Vision Research,47(21),2714–2726. 10.1016/j.visres.2007.05.007 0042-6989 17614113 PMC2048814

[b26] Reuter,M. , Kirsch,P. , & Hennig,J. (2006).Inferring candidate genes for attention deficit hyperactivity disorder (ADHD) assessed by the World Health Organization Adult ADHD Self-Report Scale (ASRS). Journal of Neural Transmission (Vienna, Austria),113(7),929–938. 10.1007/s00702-005-0366-5 0300-9564 16362639

[b55] Roberts,M. , Ashinoff,B. K. , Castellanos,F. X. , & Carrasco,M. (2018).When attention is intact in adults with ADHD. Psychonomic Bulletin & Review,25(4),1423–1434. 10.3758/s13423-017-1407-4 1069-9384 29181782 PMC5971124

[b1] Rolfs,M. (2009).Microsaccades: Small steps on a long way. Vision Research,49(20),2415– 2441. 10.1016/j.visres.2009.08.010 0042-6989 19683016

[b50] Salthouse,T. A. (2012).Psychometric properties of within-person across-session variability in accuracy of cognitive performance. Assessment,19(4),494–501. 10.1177/1073191112438744 1073-1911 22389243 PMC3632781

[b48] Saville,C. W. N. , Pawling,R. , Trullinger,M. , Daley,D. , Intriligator,J. , & Klein,C. (2011).On the stability of instability: Optimising the reliability of intra-subject variability of reaction times. Personality and Individual Differences,51(2),148–153. 10.1016/j.paid.2011.03.034 0191-8869

[b49] Saville,C. W. N. , Shikhare,S. , Iyengar,S. , Daley,D. , Intriligator,J. , Boehm,S. G. , Feige,B. , & Klein,C. (2012).Is reaction time variability consistent across sensory modalities? Insights from latent variable analysis of single-trial P3b latencies. Biological Psychology,91(2),275–282. 10.1016/j.biopsycho.2012.07.006 0301-0511 22835518

[b44] Schönbrodt,F. D. , & Perugini,M. (2013).At what sample size do correlations stabilize? Journal of Research in Personality,47(5),609–612. 10.1016/j.jrp.2013.05.009 0092-6566

[b18] Seli,P. , Smallwood,J. , Cheyne,J. A. , & Smilek,D. (2015).On the relation of mind wandering and ADHD symptomatology. Psychonomic Bulletin & Review,22(3),629–636. 10.3758/s13423-014-0793-0 1069-9384 25561417

[b19] Shaw,G. A. , & Giambra,L. (1993).Task‐unrelated thoughts of college students diagnosed as hyperactive in childhood. Developmental Neuropsychology,9(1),17–30. 10.1080/87565649309540541 8756-5641

[b27] Singer,J. L. , & Antrobus,J. S. (1963).A Factor-Analytic Study of Daydreaming and Conceptually-Related Cognitive and Personality Variables. Perceptual and Motor Skills,17(1),187–209. 10.2466/pms.1963.17.1.187 0031-5125 14045737

[b51] Steinman,R. M. (1966).Effect of target size, lumi-nance, and color on monocular fixation. JOSA,55(9),1158–1164. 10.1364/JOSA.55.001158

[b2] Thome,J. , Ehlis,A.-C. , Fallgatter,A. J. , Krauel,K. , Lange,K. W. , Riederer,P. , Romanos,M. , Taurines,R. , Tucha,O. , Uzbekov,M. , & Gerlach,M. (2012).Biomarkers for attention-deficit/hyperactivity disorder (ADHD). A consensus report of the WFSBP task force on biological markers and the World Federation of ADHD. The World Journal of Biological Psychiatry: The Official Journal of the World Federation of Socie-ties of Biological Psychiatry.,13(5),379– 400. 10.3109/15622975.2012.690535 1814-1412 22834452

[b4] Unsworth,N. , Robison,M. K. , & Miller,A. L. (2019).Individual differences in baseline oculometrics: Examining variation in baseline pupil diameter, spontaneous eye blink rate, and fixation stability. Cognitive, Affective & Behavioral Neuroscience,19(4),1074– 1093. 10.3758/s13415-019-00709-z 1530-7026 30888645

[b7] Valsecchi,M. , Betta,E. , & Turatto,M. (2007).Visual oddballs induce prolonged microsaccadic inhibition. Experimental Brain Research,177(2),196–208. 10.1007/s00221-006-0665-6 0014-4819 16951959

[b36] Watson,D. , Clark,L. A. , & Tellegen,A. (1988).Development and validation of brief measures of positive and negative affect: The PANAS scales. Journal of Personality and Social Psychology,54(6),1063–1070. 10.1037/0022-3514.54.6.1063 0022-3514 3397865

[b46] Wetzels,R. , & Wagenmakers,E.-J. (2012).A default Bayesian hypothesis test for correlations and partial correlations. Psychonomic Bulletin & Review,19(6),1057–1064. 10.3758/s13423-012-0295-x 1069-9384 22798023 PMC3505519

[b30] Whiteside,S. P. , & Lynam,D. R. (2001).The Five Factor Model and impulsivity: Using a structural model of personality to understand impulsivity. Personality and Individual Differences,30(4),669–689. 10.1016/S0191-8869(00)00064-7 0191-8869

[b54] Willcutt,E. G. , Doyle,A. E. , Nigg,J. T. , Faraone,S. V. , & Pennington,B. F. (2005).Validity of the executive function theory of attention-deficit/hyperactivity disorder: A meta-analytic review. Biological Psychiatry,57(11),1336–1346. 10.1016/j.biopsych.2005.02.006 0006-3223 15950006

[b33] Winterstein,B. P. , Silvia,P. J. , Kwapil,T. R. , Kaufman,J. C. , Reiter-Palmon,R. , & Wigert,B. (2011).Brief assessment of schizotypy: Developing short forms of the Wisconsin Schizotypy Scales. Personality and Individual Differences,51(8),920–924. 10.1016/j.paid.2011.07.027 0191-8869

